# Antihypertensive drug class and impaired fasting glucose: a risk association study among Chinese patients with uncomplicated hypertension

**DOI:** 10.1186/1472-6904-8-6

**Published:** 2008-09-10

**Authors:** Martin CS Wong, Johnny Y Jiang, H Fung, Sian Griffiths, Stewart Mercer

**Affiliations:** 1 School of Public Health and Department of Community and Family Medicine, Faculty of Medicine, Chinese University of Hong Kong, Hong Kong China; 2 Hospital Authority, Hong Kong SAR. 11/F, clinical sciences building, Prince of Wales Hospital, Shatin, NT, Hong Kong China; 3 General Practice and Primary Care, Division of Community-based sciences, Faculty of Medicine, University of Glasgow, Glasgow, G12 9LX, UK

## Abstract

**Background:**

There is a scarcity of studies addressing the factors associated with impaired fasting glucose in Chinese patients with uncomplicated hypertension. We included 1,218 patients newly prescribed a single antihypertensive drug in the public primary healthcare setting in Hong Kong, where their fasting glucose levels were measured 6–7 weeks after the first-ever antihypertensive prescription.

**Methods:**

The odds ratios of having above borderline (≥ 6.1 mmol/l) and adverse (≥ 7.0 mmol/l) glucose levels, respectively, were studied according to patient age, gender, socioeconomic status, clinic types and antihypertensive drug classes by multivariable regression analyses.

**Results:**

The fasting glucose levels were statistically similar (p = 0.786) among patients prescribed thiazide diuretics (5.48 mmol/l, 95%, 5.38, 5.59), calcium channel blockers (5.46 mmol/l, 95% C.I. 5.37, 5.54), β-blockers (5.42 mmol/l, 95% C.I. 5.34, 5.51) and drugs acting on the renin angiotensin system (RAS) [5.41 mmol/l, 95% C.I. 5.20, 5.61]. Multivariate analyses reported no significant associations between antihypertensive drug class and impaired fasting glucose. Elderly patients and male gender were significantly more likely to present with above borderline and adverse readings respectively.

**Conclusion:**

Clinicians should be aware of the increased risk of impaired fasting glucose in these groups, and use of thiazides should not in itself deter its use as a first-line antihypertensive agent among ethnic Chinese patients.

## Background

Hypertension was estimated to be present in at least 73% of citizens in the United States who have diabetes [1]. Many people were diagnosed with hypertension prior to diabetes [2], and a significant number of hypertensive patients have diabetes gone unrecognized [3]. Diabetes was reported to impose a two-fold or greater risk for cardiovascular morbidity and mortality [4] and together with hypertension this risk increased dramatically [5]. The co-existence of diabetes and hypertension are associated with greater degrees of arterial stiffness [6] which leads to earlier rises in systolic and pulse pressures [7].

The importance of early detection of diabetes among hypertensive patients has been highlighted in the guidelines of the American Diabetes Association [8] and the seventh report of the Joint National Committee [9], which called for screening for diabetes at the time of diagnosing hypertension and repeating screening annually.

There has been evidence that thiazide diuretics could precipitate overt diabetes [10] and it was well recognized in authoritative medical textbooks [11] that diuretics are "contraindications" in prescribing for maturity-onset diabetic patients. However, recent debate arose as to the best first-line antihypertensive in diabetic patients. For instance, diuretics were labeled as the least expensive and most effective agent, and "... should be the first line treatment almost everyone with hypertension, including patients with diabetes..." [12]. In addition, studies on the relationship between antihypertensive drug classes and hyperglycemia among ethnic Chinese patients were scarce. Local surveys among Chinese doctors reported that the majority chose thiazide diuretics as the best first-line agent for patients newly diagnosed with uncomplicated hypertension [13] but most changed their choice to Angiotensin Converting Enzyme inhibitors when diabetes was co-existing [14] due to physicians' concerns that impaired fasting glucose could be a significant side effect of thiazide diuretics. To our knowledge there have been no studies evaluating the association of various antihypertensive drug classes with the development of impaired fasting glucose or diabetes among ethnic Chinese patients.

The present study examined the factors associated with elevated fasting glucose in Chinese patients with uncomplicated hypertension newly prescribed a single antihypertensive prescription.

## Methods

### Data source

The Clinical Data Analysis and Reporting System (CDARS) of the Hospital Authority, Hong Kong consists of all patient records in public, primary health sector visits since 2000. It captures patients' demographics, clinical information including diagnoses, prescription details and laboratory investigation results in every out-patient consultation. One of the major functions of CDARS is research [15]. It is the single portal of information entry for all physicians practicing in the public sector in Hong Kong, and is a comprehensive record which allows cross-referencing when patients visit a different clinic in the public health sector. Also, it is the policy of all public clinics in Hong Kong to have their drug prescription details doubly checked by pharmacist professionals and any amendments were also recorded in the electronic patient record. In addition to these good practices this database has been evaluated and found a high level of completeness with respect to demographic data (100%) and prescription details (99.98%) [16]. The present study included populations residing in the New Territory East Cluster (NTEC) of Hong Kong, which has a population of around 1.3 million. This cluster is further divided into 3 separate regions, namely Shatin, Tai-Po and the North District, from the most urbanized to the most rural regions respectively. Their median monthly household incomes in 2006 were US$2,510, US$2,338 and US$2,078 for these three regions respectively, compared to the Hong Kong-wide figure of US$2,240. These three regions have similar median ages (38–39 years), comparable with the median age of 39 years for Hong Kong [17]. This study gained approval from the Survey and Behavioural Research Ethics Committee, Chinese University of Hong Kong.

### Subjects

Patients newly attended the public primary care practice, coded by physicians with International Classification of Primary Care (ICPC) K86 [uncomplicated hypertension] and who were prescribed at least one antihypertensive agent as the first-ever antihypertensive pharmacotherapy in the public sector during the study period of January 2004 to June 2007 were included. Only patients who had their fasting glucose levels checked 4 to 16 weeks after the first-ever antihypertensive prescription were included. Exclusion criteria included subjects having concomitant cardiovascular factors or clinical conditions which could confound the prescription choice of antihypertensive drugs, identified by the respective ICPC codes as follows: complicated hypertension (K87), diabetes mellitus (T90), impaired glucose tolerance (T91), gout (T92), lipid disorder (T93), stroke and cerebrovascular accident (K90 and K91), ischemic heart diseases (K74, K76), acute myocardial infarction (K75), heart failure and heart diseases (K77, K84, K99), chronic obstructive pulmonary diseases, asthma (R79, R95, R96), and urological conditions (U14, U88, Y85, U78, Y79). Also, patients discontinued or switched their drug prescription before fasting glucose was measured, or prescribed at least one oral hypoglycemic agents or insulin preparations during the study period were excluded from the analysis.

Each patient was classified into one drug group according to the antihypertensive drug class prescribed. These include BBs, thiazide diuretics, Calcium Channel Blockers (CCBs), drugs acting on the rennin angiotensin system (RAS) and others (including α-blockers, potassium-sparing diuretics, vasodilators and combination therapy).

### Outcomes and Covariates

The proportions of patients having desirable (≤ 6.0 mmol/l), borderline (6.1–6.9 mmol/l) and adverse (≥ 7.0 mmol/l) levels of fasting glucose levels were compared among the various drug classes. Plasma glucose from blood collected after an overnight fast of 8 to 12 hours was assayed enzymatically with commercial reagents (Abbott Laboratory Limited). Independent predictors and covariates of elevated fasting glucose levels include patient age, gender, patients' payment status (fee waivers vs. payers; each consultation costs US$5.77 including prescription and investigation fees), district of residence, type of clinics attended, namely General Out Patient Clinics (GOPCs), Family Medicine Specialist Clinics (FMSCs) and Staff Clinics (SCs), and the antihypertensive drug classes prescribed (thiazide diuretics, BB, CCB, RAS).

### Statistical Analysis

All the data were entered into the Statistical Package for Social Sciences (SPSS) version 13.0. A descriptive analysis was performed and the proportions of patients having desirable, borderline and adverse lipid results were compared by χ^2 ^tests of homogeneity. Student's t-tests were used to compare continuous variables, except the time periods from antihypertensive prescription to blood measurement where Kruskal Wallis test was used for the skewed distribution. The crude odds ratios of each drug class having above borderline (≥ 6.1 mmol/l) and adverse (≥ 7.0 mmol/l) glucose readings were examined respectively using bivariate analysis. This is followed by two separate unconditional binary logistic regression analyses for above borderline and adverse glucose readings using all the potential associated factors and covariates as independent variables. The enter method was adopted as the regression selection procedure, and drug classes included thiazide diuretics, BB and CCB with RAS as a reference group. All p values were two-sided, and p < 0.05 were regarded as statistically significant.

## Results

### Patient characteristics

There were 1,218 eligible patients (Table 1). Overall the mean age was 60.39 years (95% C.I. 59.67, 61.11 years), with females comprising 58.6% of the sample. The median period between the first-ever antihypertensive drug prescription and checking of fasting glucose was 7 weeks and these periods were statistically similar among all the drug groups (p = 0.054) (Table 1). The average age of patients varied significantly across different drug groups (p < 0.001), thought the size of the differences were relatively small (Table 1). Gender differences according to the type of drug prescribed were also evident (p = 0.008). Socio-economic status (with payment status as a proxy measure) of patients did not vary significantly across drug classes (p = 0.415). Most of them lived in more rural areas (29.6%, 11.3%, 51.3% and 7.8% living in Shatin, Taipo, North district and other regions respectively), a finding which reflects that less affluent regions have higher proportion of patients suffering from uncomplicated hypertension only as the single disease entity without other clustering of cardiovascular (CVS) factors. The most frequently attended clinics were GOPCs (86.5%), followed by FMSCs (10.1%) and staff clinics (3.4%), statistically different across antihypertensive drug classes (p < 0.001).

**Table 1:** Patient characteristics (N = 1,218)

	β-blockers (n = 307)	Thiazide (n = 196)	CCBs (n = 322)	RAS (n = 55)	Others* (n = 338)	p value
Age (years)						
<50	85 (27.7)	38 (19.4)	74 (23.0)	9 (16.4)	58 (17.2)	<0.001
50–59	113 (36.8)	62 (31.6)	87 (27.0)	22 (40.0)	84 (24.9)	
60–69	59 (19.2)	51 (26.0)	70 (21.7)	11 (20.0)	77 (22.8)	
≥ 70	50 (16.3)	45 (23.0)	91 (28.3)	13 (23.6)	119 (35.2)	
Mean Age	56.96	60.14	60.80	60.02	63.30	<0.001
(SD)	(11.67)	(11.37)	(13.21)	(12.83)	(13.42)	
(95% CI)	(55.651,58.27)	(58.54, 61.74)	(59.36, 62.25)	(56.55, 63.49)	(61.87, 64.74)	
Gender (no./%)						
Female	200 (65.1)	124 (63.3)	168 (52.2)	33 (60.0)	189 (55.9)	0.008
Male	107 (34.9)	72 (36.7)	154 (47.8)	22 (40.0)	149 (44.1)	
Payment						
Fee-waivers	107 (34.9)	68 (34.7)	107 (33.2)	17 (30.9)	96 (28.4)	0.415
Payers	200 (65.1)	128 (65.3)	215 (66.8)	38 (69.1)	242 (71.6)	
District of residence						
Shatin	85 (27.7)	44 (22.4)	84 (26.1)	12 (21.8)	135 (39.9)	<0.001
Taipo	31 (10.1)	13 (6.6)	52 (16.1)	5 (9.1)	37 (10.9)	
Northern	161 (52.8)	127 (64.8)	169 (52.5)	34 (61.8)	134 (39.6)	
Others	30 (9.4)	12 (6.1)	17 (5.3)	4 (7.3)	32 (9.5)	
Service type						
General	264 (86.0)	179 (91.3)	288 (89.4)	48 (87.3)	275 (81.4)	<0.001
FMSC	29 (9.4)	7 (3.6)	27 (8.4)	4 (7.3)	56 (16.6)	
Staff clinic	14 (4.6)	10 (5.1)	7 (2.2)	3 (5.5)	7 (2.1)	
Glucose Profile	5.42	5.48	5.46	5.41	5.60	0.786
(mean ± 95% CI)	(5.34, 5.51)	(5.38, 5.59)	(5.37, 5.54)	(5.20, 5.61)	(5.52, 5.68)	
Period from drug prescription to blood taking (weeks)	7.43	7.00	7.79	6.71	8.00	0.054**
Median (IQR)	(5.86, 10.00)	(5.18, 8.96)	(5.57, 10.32)	(5.00, 10.00)	(6.00, 12.00)	

(CCB: Calcium channel blockers; RAS: drugs acting on the rennin angiotensin system, IQR: Inter-quartile Ranges)

*Others refer to drug classes including α-blockers, polytherapy, combination therapy, and Misc classes

**Refers to comparison among four drug groups: β-blockers, thiazide diuretics, CCB, RAS)

### Patterns of fasting glucose profiles by antihypertensive drug group

Among the four major antihypertensive drug classes, the mean glucose level was 5.45 mmol/l (95% C.I. 5.41, 5.49). Thiazide users had the highest absolute levels of fasting glucose (5.48 mmol/l), followed by CCB (5.46 mmol/l), BB (5.42 mmol/l) and RAS (5.41 mmol/l). However these apparent differences in glucose parameters between drug groups were all statistically insignificant (p = 0.786) (Table 1).

The overall proportions of patients having desirable, borderline and adverse fasting glucose were 83.2%, 13.6%, and 3.2% respectively (Table 2). Among the four drug classes, the proportions of patients having borderline or adverse fasting glucose levels were highest among RAS users (16.0%), followed by BB (15.7%), thiazide diuretics (15.0%) and CCB (13.0%) (Table 2), but these differences were statistically insignificant (p = 0.849).

**Table 2:** Patterns of glucose profiles by drug group (No./%)

	β-blockers	Thiazide	CCBs	RAS	Others	P (χ^2^)
Glucose (mmol/l)	(n = 280)	(n = 180)	(n = 291)	(n = 50)	(n = 296)	
Desirable (≤ 6.0)	236 (84.3)	153 (85.0)	253 (86.9)	42 (84.0)	229 (77.4)	0.849
Borderline (6.1–6.9)	35 (12.5)	22 (12.2)	28 (9.6)	5 (10.0)	59 (19.9)	
Adverse (≥ 7.0)	9 (3.2)	5 (2.8)	10 (3.4)	3 (6.0)	8 (2.7)	

(CCB: Calcium channel blockers; RAS: drugs acting on the rennin angiotensin system.

Glucose levels for desirable: ≤ 6.0 mmol/l, borderline: 6.1–6.9 mmol/l; adverse: ≥ 7.0 mmol/l)

### Factors associated with impaired fasting glucose

From bivariate analysis between drug class prescribed and the likelihood of patients having adverse fasting glucose readings, CCB users were significantly less likely to present with above borderline fasting glucose (OR 0.604, 95% C.I. 0.390, 0.933, p = 0.022) when compared with other drug classes (Table 3). Otherwise all other comparisons among the four drug classes were statistically similar (p = 0.278 to 0.986). However, after controlling for confounding variables by means of multivariate logistic regression, all drug classes were statistically similar in presenting with above borderline (p = 0.549 to 0.819) and adverse (p = 0.334 to 0.441) glucose readings.

**Table 3:** The crude hazard ratios of having above borderline and adverse glucose profiles for each antihypertensive drug group

	Bivariate Analysis Crude Odds Ratios for above borderline results (95% C.I.) (≥ 6.1 mmol/l)	p value	Bivariate Analysis Crude Odds Ratios for adverse results (95% C.I.) (≥ 7.0 mmol/l)	p value
(1). β-blockers	0.881 (0.586, 1.323)	0.540	0.993 (0.459, 2.150)	0.986
(2). Thiazide Diuretics	0.860 (0.530, 1.397)	0.543	0.828 (0.316, 2.168)	0.700
(3). CCB	0.604 (0.390, 0.933)	0.022	1.043 (0.494, 2.204)	0.911
(4). RAS	0.720 (0.280, 1.850)	0.493	1.944 (0.572, 6.607)	0.278

(CCB: Calcium channel blockers; RAS: drugs acting on the rennin angiotensin system. Glucose levels for desirable: ≤ 6.0 mmol/l, above borderline: ≥ 6.1 mmol/l; adverse: ≥ 7.0 mmol/l. The desirable group was used as the reference)

When compared to young age (<50 years), older patients ≥ 70 years were 2.103 times (95% C.I. 1.051, 4.207, p = 0.036) more likely to present with above borderline glucose levels (Table 4). Age was not significantly associated with adverse glucose readings. While female patients were similarly likely to have above borderline glucose readings when compared with male patients (p = 0.102), they were significantly less likely to have adverse readings (OR 0.223, 95% C.I. 0.074, 0.674, p = 0.008). Payment status was not significantly associated with above borderline and adverse glucose levels. Patients living in the North district, a region with the lowest income level among all districts, were significantly less likely to have adverse glucose levels (OR 0.311, 95% C.I. 0.115, 0.841, p = 0.021) than residents in Shatin.

**Table 4:** Factors associated with hyperglycemia with above borderline and adverse readings*

	Above Borderline readings		Adverse readings	
	
	Odds Ratios (95% CI)	p	Odds Ratios (95% CI)	p
Age (years)				
<50	1.0 (ref)		1.0 (ref)	
50–59	1.249 (0.648, 2.408)	0.506	1.776 (0.533, 5.919)	0.349
60–69	1.344 (0.654, 2.759)	0.421	0.784 (0.167, 3.685)	0.758
≥ 70	2.103 (1.051, 4.207)	0.036	1.359 (0.372, 4.962)	0.643
Gender				
Male	1.0 (ref)		1.0 (ref)	
Female	1.464 (0.927, 2.313)	0.102	0.223 (0.074, 0.674)	0.008
Payment status				
Fee waiver	1.0 (ref)		1.0 (ref)	
Fee payers	1.328 (0.789, 2.236)	0.286	0.759 (0.320, 1.798)	0.530
District of residence				
Shatin	1.0 (ref)		1.0 (ref)	
Taipo	0.967 (0.433, 2.156)	0.934	1.676 (0.588, 4.778)	0.334
North	0.707 (0.392, 1.273)	0.248	0.311 (0.115, 0.841)	0.021
Others	0.909 (0.363, 2.279)	0.839	N/A	N/A
Clinics				
GOPC	1.0 (ref)			
FMSC	0.652 (0.234, 1.818)	0.414	0.299 (0.036, 2.507)	0.266
Staff clinics	2.071 (0.717, 5.984)	0.179	0.614 (0.066, 5.732)	0.668
Drug class				
RAS	1.0 (ref)		1.0 (ref)	
β-blockers	1.360 (0.497, 3.721)	0.549	0.518 (0.125, 2.151)	0.365
Thiazide	1.252 (0.443, 3.537)	0.672	0.466 (0.099, 2.190)	0.334
CCB	0.888 (0.320, 2.463)	0.819	0.575 (0.140, 2.353)	0.441

(C.I: confidence intervals, GOPC: General Out-patient clinics, FMSC: Family Medicine Specialist Clinics. RAS: drugs acting on the renin angiotensin system; CCB: Calcium channel blockers.

*Above borderline readings were defined as fasting glucose ≥ 6.1 mmol/l; adverse readings as fasting glucose ≥ 7.0 mmol/l. All adjusted Odds Ratio were adjusted for age, gender, payment status, district of residence, service types of attended clinics and other drug classes as listed in table

N/A: No patients presented with adverse readings)

Patients attending different types of clinics were found to have similar odds of having above borderline (p = 0.179 to 0.414) and adverse fasting glucose levels (p = 0.266 to 0.668) (Table 4).

## Discussion

### Major Findings

In the present study, we examined the factors associated with impaired fasting glucose among Chinese hypertensive patients prescribed a single antihypertensive agent. No significant associations were found between antihypertensive drug class and different extents of impaired fasting glucose. Elderly patients (≥ 70 years) were more likely to have above borderline fasting glucose levels while female patients were less likely to have adverse glucose readings. Patients residing in rural areas were less likely to present with adverse glucose readings.

### Interpretations of findings and relationship to published literature

Thiazide diuretics could increase insulin resistance [18], affect glucose utilization [2], precipitate overt diabetes [10] and worsen diabetes control [19] but the average rise in blood glucose levels in long term resistance trials in diuretic-treated patients was small compared with placebo subjects. Also in large prospective trials unfavorable clinical outcomes have not been reported, and the increase in cumulative incidence of new-onset diabetes is by approximately 0.6% only [20-25].

From the ALLHAT study a small increase in serum glucose and a minor rise (3.5%) in the absolute risk of developing new-onset diabetes was reported in the thiazide group (11.6%) versus lisinopril (8.1%), but the frequency of CVS events was found to be similar between the two drug classes in a five year period [26]. This finding is in echo with the results from the Controlled Onset Verapamil Investigation of Cardiovascular End Points (CONVINCE) trial where diabetic cohorts receiving verapamil, thiazides or β-blockers had similar CVS outcomes [27]. In addition, in a large prospective cohort study of 12,500 hypertensive patients [28] no increase in the incidence of diabetes was noted with therapeutically low doses equivalent to 12.5 mg of hydrochlorothiazide.

Another notable finding from ALLHAT revealed that diabetic subjects who received thiazide diuretics had coronary heart disease outcomes equal to diabetic subjects who received ACEIs (namely lisinopril or amlodipine), yet suffered from fewer strokes or episodes of heart failure than the lisinopril group. In both diabetic and non-diabetic cohorts, less incidence of heart failure was reported among patients treated with thiazide diuretics than with amlodipine [26]. The observation that thiazide use in diabetic patients could be favorable is also supported by the majority of studies where diabetic patients had fewer CVS outcomes than non-diabetic patients when both groups were prescribed thiazides [29]. Also patients receiving a thiazide (chlorthalidone) in the SHEP trial, although having an increased incidence of diabetes, did not have a higher rate of CVS events after an average of 14.3 years follow-up [30].

However, it should be noted that current literatures are still mixed in the opinions of using thiazides in diabetic patients, where "future troubles should be recognized" [31] particularly in newly diagnosed diabetic patients who may have increased risk of heart attack [32]. Although studies have demonstrated superior efficacy of drugs acting on RAS in preventing new-onset type 2 diabetes, namely captopril [33], ramipril [34] and losartan [35], thaizides were found to be necessary to reach blood pressure goals in diabetic patients. The protective effect of RAS on occurrence of diabetes has been postulated to be potassium elevation which has been recognized to play a crucial role in preventing diabetes [36,37]. Combined use of RAS and thiazide diuretics have been recommended in hypertensive patients with family history of diabetes or components of metabolic syndrome like obesity, especially in patients with stage 2 hypertension [2].

The present study has reported no association of antihypertensive drug class with any degrees of impaired fasting glucose, and is therefore compatible with evidence favoring the use of thiazide diuretics in uncomplicated hypertension applicable also to ethnic Chinese patients.

It was well recognized that ageing is associated with impaired beta cell repair, insulin resistance and hence impaired fasting glucose [38] which is consistent with our study findings reporting ageing as positively associated with above borderline glucose levels. The relative risks of diabetes for women have been higher than men in all ages [39], and women were also more likely to have abnormal glucose regulation [40]. The present study, on the contrary, demonstrated that female is at lower risk of having adverse glucose readings.

Hyperglycaemia is a concentration-dependent predictor of long-term mortality after a coronary event, and this predictive value is stronger in men than women [41]. But risk of coronary heart disease in women may be elevated at a lower glucose level than for men [42], consistent with other findings that diabetes was reported as a greater risk factor for cardiovascular diseases in women than in men [43,44]. Therefore in the present study, the lower odds of having impaired fasting glucose in women than men should be interpreted in the light of the greater risk of incidence of CVS diseases among diabetic women than diabetic men.

Patients residing in more rural areas, namely the North district, may have more healthy dietary lifestyles. No studies have evaluated the association between socioeconomic status and elevated fasting glucose, and this area remains to be explored.

### Strengths and weaknesses of current study

The present study adopted an observational design including only antihypertensive drug naïve patients newly received an antihypertensive agent without oral hypoglycemic agents or insulin use, concomitant cardiovascular disorders or other medical conditions which could confound choice of prescriptions. The periods where glucose levels were checked after the prescription were approximately 7 weeks which were statistically similar among the four drug groups. In conjunction with our fairly large sample size we believed this study reflects real-world clinical practice. Also our patients had similar sociodemographic characteristics compared to the entire population of Hong Kong [21] making it more likely that our findings are generalizable.

However, some limitations of this study should be discussed. First, we do not have a baseline glucose profile which could act as reference levels for comparisons of levels before and after drug prescriptions, and this study only evaluated a short time frame of approximately seven weeks, a period which might not suffice metabolic effects to take place. Also, we regarded the absence of ICPC coding as meaning the absence of the relevant condition, but in a previous validation study of this clinical database [16] it was shown that patients coded with K86 (uncomplicated hypertension) only were likely to be suffering from uncomplicated hypertension as the single disease entity without other concomitant medical conditions. In addition, age, gender and district of residence were associated with either above borderline or adverse glucose readings (but not both), which might weaken the conclusion drawn. Some confounders like smoking and risk factors for developing impaired glucose tolerance like obesity could not be controlled for due to lack of relevant data, and the absence of oral glucose tolerance tests would mean the diagnosis of impaired glucose tolerance could not be ascertained. Finally, our study cannot prove causative relationships between the predictor variables and glucose profiles, which could only be addressed by a prospective cohort design.

### Implications for policy and practice

This study evaluated age, gender, socioeconomic factors, types of clinic services and the antihypertensive drug classes as associated factors of impaired fasting glucose in Chinese patients with uncomplicated hypertension. Physicians may practice higher caution in managing hypertensive patients with these associated factors at higher risk of impaired fasting glucose, implying a greater need for more regular screening of glucose profiles, especially among older patients, males, and those residing in more urbanized areas. The antihypertensive drug classes were not shown to be associated with impaired glucose levels, nor were they associated with drug discontinuation [45,46], switching [46] or dyslipidemia [47] as reported in previous studies. The use of thiazide as a first-line agent in patients with uncomplicated hypertension could be justified in ethnic Chinese according to recommendations from most international guidelines.

## Conclusion

We examined the independent factors associated with the presentation of impaired fasting glucose among 1,218 hypertensive patients newly prescribed a single antihypertensive agent. Age, gender and district of residence were significant independent predictors of impaired fasting glucose among Chinese hypertensive patients while antihypertensive drug classes were not associated with elevated glucose readings. Physicians could use these independent factors as a reference for more meticulous monitoring of glucose levels among hypertensive Chinese patients presented with these associated factors.

## Competing interests

The authors declare that they have no competing interests.

## Authors' contributions

MCSW has contributed to the conception, design, data acquisition, analysis of data and prepared the first draft of this paper. JYJ contributed to refining the methodology, data analysis, and intellectual input to data interpretation; HF, SG and SWM contributed to amendment of initial study design, analysis of data, intellectual input and critically revising the manuscript. All authors read and approved the final manuscript. SWM is guarantor for the study.

## Pre-publication history

The pre-publication history for this paper can be accessed here:


